# Some Observations on Injuries of the Brain

**Published:** 1821-03

**Authors:** C. C. Wallis

**Affiliations:** Member of the Royal College of Surgeons, and Licentiate of the Society of Apothecaries, of London.


*Some Observations on Injuries of the Brain.* By C. C. Wallis, Member of the Royal College of Surgeons, and Licentiate of the Society of Apothecaries, of London.

FACTS which have a tendency to elucidate pathological obscurities, are at all times worthy of our best considerations; and it is therefore to be regretted that rare occurrences in surgical practice are not more minutely attended to, or, rather, that their knowledge is not more generally diffused. This circumstance may arise from a want of zeal on the part of the practitioner,—from too fastidious conceptions of his own capacity,—from dread of public criticism,—or from his time being too exclusively required by other more immediate and less avoidable professional duties. Every member of the profession, however, were he actuated by so desirable a spirit, and that spirit not opposed by the intervention of insuperable barriers, would unquestionably have frequent opportunities of contributing largely to the advancement of science; and it would then be imperative on him to give all possible publicity to every extraordinary or uncommon case that might fall under his cognizance.

Accidents and diseases occurring to the brain are not the most unfrequent affections that claim the assistance of surgery, and are at all times subjects demanding the practitioner's mature and deliberate investigation. Much obscurity for a long time prevailed respecting the effects of injuries of this organ; and the uncertainty of the diagnosis of compression, concussion, &c. was a reason for many of the French surgeons (among them Bichât and Dessault) for renouncing the trephine. Definite discriminations and symptoms characteristic of each state have, however, been lately made with much success by our justly-celebrated countryman, Mr. Abernethy.*

Injuries of the head are, generally speaking, dangerous from the undue pressure produced on the brain by depression of bone; and extravasation of blood, or the formation of matter, within the cranium; each of which causes will finally produce the same symptoms,—viz. insensibility, weak pulse, dilated and immovable pupil, stertorous respiration, &c. and can be relieved only by the timely application of the trephine. Before, however, such an application is made, it is of the utmost moment to know which of the several causes alluded to is exerting the baneful influence we are called upon to remove.

Depression of bone, then, is characterized from either of the other two states, by the symptoms supervening immediately after the infliction of the injury; by there having been no interval of sense, nor time sufficient for the completion of suppuration.

It will be necessary, also, to distinguish depression of bone from a state of an equally alarming nature—concussion. This kind of injury is denoted by a sudden stunning or insensibility; the patient is cold, the pulse slow and intermitting, and respiration scarcely perceptible. In proportion as the stupefaction goes off, increase of pulse and respiration take place, the pupil is contracted, the patient is sick, and vomits. If roused, he

* Surgical Works, vol. ii.

will answer peevishly, and then fall asleep again. As the disease advances, febrile action is manifested, and phrenitis rapidly ensues.

I have been led to the foregoing remarks by a recent occurrence in this neighbourhood, which will undergo judicial investigation at the ensuing assizes. It was a case wherein the operation of trephining was performed about a fortnight after the receipt of external violence; and it was stated in evidence at the coroner's inquest, that the patient followed his accustomed bodily labour for *five days* after the infliction of the injury; but that, on opening the head after death, the skull was found to be extensively fractured, and that several spiculæ of bone had actually been driven into, and produced disorganization of, the substance of the brain itself, although *fourteen days* had elapsed before the usual symptoms manifested themselves.

These facts present forcible contradictions to the doctrines hitherto promulged on this interesting and important topic; and society will be benefitted, and myself much obliged, to any of your readers who will explain this apparently irreconcilable contrariety.*

*Langport; January 8th*, 1821.

---

* We insert this paper because it contains an useful lesson that may be repeated from time to time, without disadvantage, to many practitioners. The records of Surgery, and especially *ex professo* treatises on the subjects of Medical Jurisprudence, abound with cases analogous to that above related by Mr. WALLIS; whose query, we suspect, cannot be replied to in a satisfactory manner. Between the fatal consequences from as slight a blow on the head as that received by the girl whose case is related by Hippocrates, and the retention, for years, of a bullet in the midst of the brain without apparent harm, we commonly find many varieties of results from similar modes and immediate consequences of external violence; but, of the causes of those varieties pathologists are as ignorant as they are of the reason why one man is salivated by a grain of calomel, whilst others will take a drachm of the same mineral without such an effect being produced. Dr. MALE's Elements of Medical Jurisprudence will show the views that are to be taken of such cases as that of Mr. Wallis, in relation to jurisprudence.—EDIT.

